# Nondestructive, high-resolution, chemically specific 3D nanostructure characterization using phase-sensitive EUV imaging reflectometry

**DOI:** 10.1126/sciadv.abd9667

**Published:** 2021-01-27

**Authors:** Michael Tanksalvala, Christina L. Porter, Yuka Esashi, Bin Wang, Nicholas W. Jenkins, Zhe Zhang, Galen P. Miley, Joshua L. Knobloch, Brendan McBennett, Naoto Horiguchi, Sadegh Yazdi, Jihan Zhou, Matthew N. Jacobs, Charles S. Bevis, Robert M. Karl, Peter Johnsen, David Ren, Laura Waller, Daniel E. Adams, Seth L. Cousin, Chen-Ting Liao, Jianwei Miao, Michael Gerrity, Henry C. Kapteyn, Margaret M. Murnane

**Affiliations:** 1 STROBE Science and Technology Center, JILA, University of Colorado, Boulder, CO 80309, USA.; 2 Department of Chemistry, Northwestern University, 2145 Sheridan Road, Evanston, IL 60208, USA.; 3 Imec, Kapeldreef 75, 3001 Leuven, Belgium.; 4 Renewable and Sustainable Energy Institute (RASEI), University of Colorado, Boulder, CO 80309, USA.; 5 Department of Physics and Astronomy and California NanoSystem Institute, University of California, Los Angeles, CA 90095, USA.; 6 Department of Electrical Engineering and Computer Sciences, University of California, Berkeley, CA 94720, USA.; 7 KMLabs Inc., 4775 Walnut St. #102, Boulder, CO 80301, USA.

## Abstract

Next-generation nano- and quantum devices have increasingly complex 3D structure. As the dimensions of these devices shrink to the nanoscale, their performance is often governed by interface quality or precise chemical or dopant composition. Here, we present the first phase-sensitive extreme ultraviolet imaging reflectometer. It combines the excellent phase stability of coherent high-harmonic sources, the unique chemical sensitivity of extreme ultraviolet reflectometry, and state-of-the-art ptychography imaging algorithms. This tabletop microscope can nondestructively probe surface topography, layer thicknesses, and interface quality, as well as dopant concentrations and profiles. High-fidelity imaging was achieved by implementing variable-angle ptychographic imaging, by using total variation regularization to mitigate noise and artifacts in the reconstructed image, and by using a high-brightness, high-harmonic source with excellent intensity and wavefront stability. We validate our measurements through multiscale, multimodal imaging to show that this technique has unique advantages compared with other techniques based on electron and scanning probe microscopies.

## Teaser

A new imaging technique enables nondestructive, 3D characterization of nanostructures, their composition, and interfaces.

## INTRODUCTION

Although x-ray imaging has been explored for decades and visible-wavelength microscopy for centuries, it is only recently that the spectral region in between―the extreme ultraviolet (EUV; with wavelengths spanning ~10 to 100 nm)―has been explored for imaging nanostructures and nanomaterials. This is because high–numerical aperture (NA), high-quality optics have not been available in the EUV region of the spectrum. However, with the practical implementation of coherent EUV light sources based on high-harmonic generation (HHG), combined with coherent diffraction imaging (CDI) (*1*), EUV imaging has been shown to be competitive in terms of resolution when compared with other light-based imaging techniques (*2*–*4*). This is important because for synthesis and integration of a host of next-generation materials and nanostructures, new approaches are needed to nondestructively and routinely determine interfacial and layer structure as well as surface morphology, with sensitivity to dopant distributions and material composition. This is becoming more critical as films and devices shrink below 10 nm, where their properties are no longer well described by bulk macroscopic models and can become almost entirely geometry or interface dominated (*5*–*9*). Moreover, the functional properties of interfaces (i.e., charge, spin, and heat transport) that affect the switching energy of magnetic memory or the coherence time and operating temperature of quantum devices are very difficult to measure, especially in situ in working devices (*10*–*12*). As a result, there is a great need for nondestructive, noncontact imaging techniques that can be applied to general samples.

Imaging with EUV light has many unique advantages. It can penetrate materials that are opaque to visible light, making it possible to image buried structures and to extract depth-dependent composition (*13*). When incident at angles between grazing and ~45°, EUV light has a sufficiently high reflectivity to image most samples (*14*–*19*). Combined with the fact that the penetration depth of EUV light is sufficiently long to probe interesting structures in most materials, this makes EUV light well suited for general reflectometry applications. This is in contrast to soft/hard x-ray light at wavelengths <8 nm, which is best suited for transmission mode microscopy. Fortunately, high-brightness, coherent EUV beams can now routinely be generated via high-harmonic up-conversion of intense femtosecond lasers (*20*–*22*). The low driving laser pulse energies required for HHG―in the 10 μJ to ~mJ range―make it possible to operate at kilohertz-to-megahertz repetition rates that are ideal for applications in imaging and spectroscopy.

**Fig. 1:**
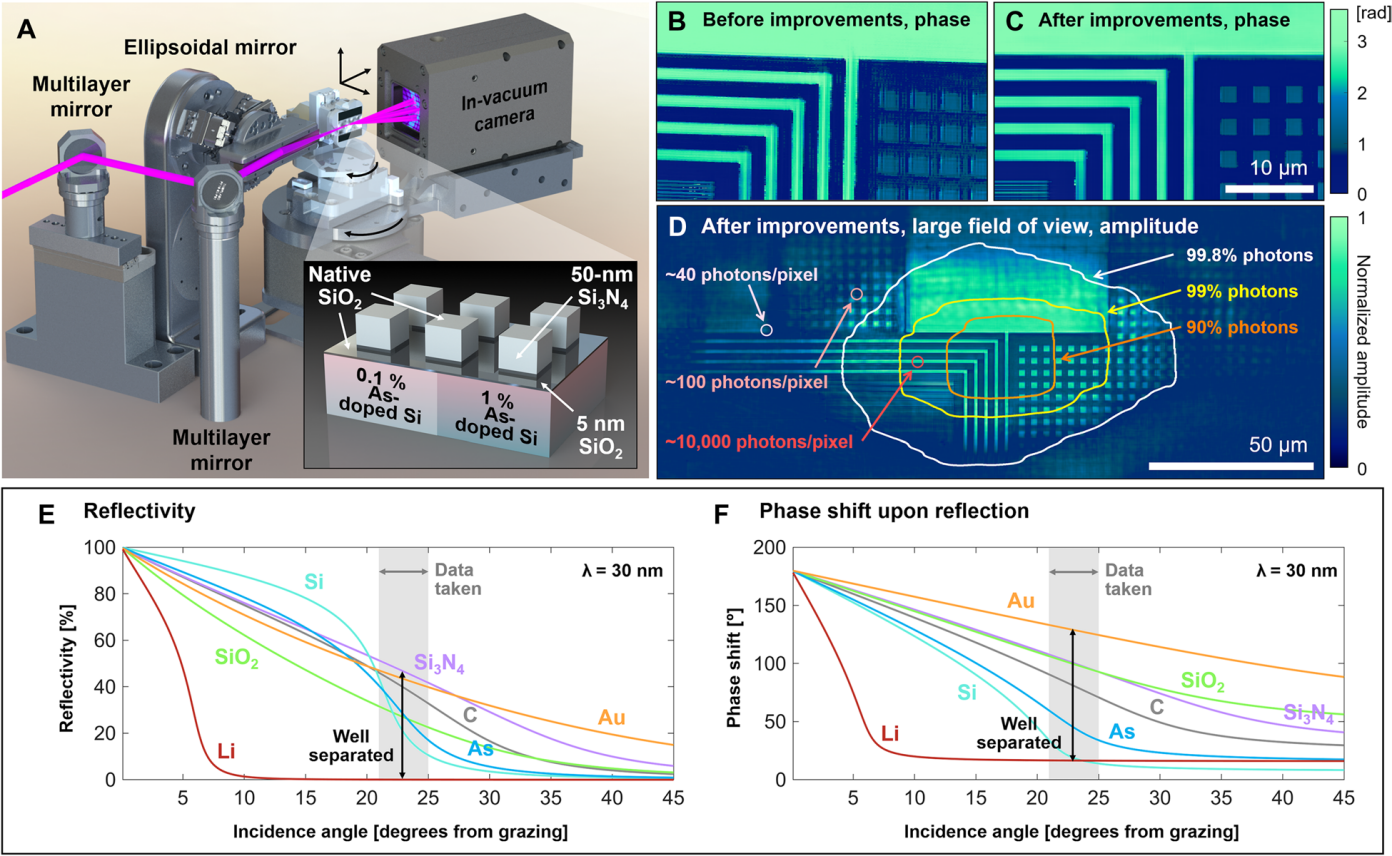
Experiment overview and nanostructure imaging. (**A**) Schematic of the amplitude- and phase-sensitive imaging reflectometer, which produces large-area, spatially and depth-resolved maps nondestructively. The incidence angle of the illumination is scanned by rotating the sample and detector in a θ-2θ configuration. The sample can also be scanned in 2D to perform ptychographic coherent diffractive imaging. Inset: Schematic representation of the imaged sample, which has SiO_2_ + Si_3_N_4_ structures patterned on As-doped regions with higher (~1%) and lower (~0.1%) peak dopant concentration. Native oxide layers (SiO_2_) are also present. (**B** and **C**) Zoom-in of EUV ptychographic phase reconstructions of the sample, (B) before and (C) after precise implementation of 3D tilted-plane correction and total variation (TV) regularization. (**D**) Entire, wide field-of-view amplitude reconstruction. Contours with corresponding labels on the right show the regions exposed to certain percentages of total photons that were incident on the sample during a single ptychography dataset. Small circles and corresponding labels on the left indicate the total number of photons that were incident on a pixel at that location over the duration of a single ptychography dataset (light incident at 30° from grazing). (**E** and **F**) Characteristic reflectivity versus angle curves for several bulk materials at 30-nm wavelength, showing the sensitivity of EUV light to material composition. The phase, measured by our reflectometer but not detected by others, can distinguish between materials even more sensitively than amplitude.

When combined with ptychographic CDI (*2*, *23*–*28*), EUV imaging can fill many current characterization gaps. In ptychography, a coherent beam of light is scanned across a sample, and the far-field diffracted intensity is collected from overlapping fields of view (FOV). An iterative phase retrieval algorithm is then used to extract quantitative images of the sample’s complex transmittance or reflectance from the collected intensity images (*29*–*31*). Recent advances in CDI are yielding stunning, high-fidelity images and transforming short-wavelength imaging capabilities (*1*, *26*, *32*–*38*). Moreover, by eliminating the need for an image-forming lens, CDI supports diffraction-limited resolution to enable high transverse resolution and axial precision at short wavelengths (*2*, *4*, *13*, *26*). Since ptychographic CDI reconstructs a sample’s full complex reflectance, its use with EUV wavelengths is well suited for phase-sensitive imaging reflectometry applications. In particular, the complex reflectance of coherent EUV light, especially the phase, is exquisitely sensitive to chemical composition, making it possible to determine sample composition uniquely when the incidence angle and/or wavelength of the beam on the sample is scanned (see Fig. 1, E and F).

Here, we demonstrate the unique advantages of coherent EUV imaging in reflection mode as a general nanoimaging technique. Our previous work has shown that EUV phase-sensitive imaging has promise for measuring many sample parameters; however, in that work, actually determining the sample parameters was a difficult-to-solve, underdetermined problem because it only used a single image of the sample (*13*). Here, we show that imaging at many angles of incidence enables us to nondestructively image nanostructures without any special sample preparation or supporting measurements from other metrologies and with unique three-dimensional (3D) compositional specificity that has not heretofore been possible. This new technique combines the excellent phase stability of coherent high-harmonic sources with the unique chemical and phase sensitivity of EUV reflectometry and state-of-the-art ptychography imaging algorithms. Several aspects were key to implementing high-fidelity phase and amplitude imaging: the ability to correct the glancing-incidence distorted diffraction patterns with very high accuracy, the use of total variation (TV) regularization (*39*) to reduce noise and artifacts in the reconstruction, the accurate self-calibration of the reflectometer, and the use of a high-brightness, high-harmonic illumination beam that is very stable in intensity, wavelength, and wavefront.

The importance of this advance is that it enables nondestructive, large-area, quantitative, 3D imaging of nanostructures and their chemical makeup, layer thicknesses, interface quality, and dopant levels. Moreover, this technique does not require any special sample preparation. The sensitivity that we achieve for some of these parameters is comparable to, or exceeds that of, other techniques that are destructive or contact-based, or that need to average over large unpatterned areas to extract some sample parameters. These include scanning transmission electron microscopy (STEM), secondary ion mass spectrometry (SIMS), and atomic force microscopy (AFM), which were used for correlative imaging in this work. In the future, by harnessing the femtosecond time resolution of EUV HHG beams, the imaging reflectometer can be enhanced further to capture charge, spin and heat transport, and link structure to function. The spatial resolution, sensitivity, and speed can also be enhanced further by using shorter-wavelength illumination, incidence angles farther from grazing, higher NA, faster detectors, and higher repetition rate drive lasers.

### Experiment setup and procedure for phase-sensitive imaging reflectometry

To implement phase-sensitive imaging reflectometry, we first record a ptychographic dataset at each incidence angle (see Fig. 1). We used five angles in this initial work; however, with increased data handling capabilities, more angles could be used to solve for more parameters or to lower the uncertainty. Next, we reconstruct an image for each angle using ptychography, giving us quantitative images of the sample’s complex reflectance at each angle. These images look similar to one another, but the features change contrast depending on their composition, as well as the wavelength and incidence angle of the illuminating beam. We then segment these images to form reflectance curves for the different sample regions, and using these, we reconstruct their depth-dependent compositions using a genetic algorithm. Last, we combine these into a representation of the sample’s topography and composition.

This phase-sensitive imaging reflectometer is illuminated by a coherent high-harmonic beam from a tabletop HHG source (modified prototype KMLabs XUUS4), at wavelengths that can range from 13 to 30 nm. A wavelength of 29.3 nm was used in this initial demonstration to use the relatively higher reflectivity of the sample at longer wavelengths: The S-polarized reflectivity of passivated Si with a native oxide layer at angles between 21° and 25° from grazing is 3 to 15%.

The test sample used in this work was custom fabricated by the Interuniversity Microelectronics Centre (Imec). The wafer is a standard 300-mm wafer that used a 65-nm node complementary metal-oxide semiconductor mask set and was doped in different regions with two different doses of As to investigate sensitivity. It had four different types of regions, as shown in the inset of Fig. 1A. The sample is on a silicon substrate that has been selectively doped in some regions to ~1% (atomic %, corresponding to 5 × 10^20^ atoms/cm^3^) at an implantation dose of 10^15^ atoms/cm^2^ and then further uniformly doped with a lower dose of 10^14^ atoms/cm^2^. Subsequently, SiO_2_ and Si_3_N_4_ structures were patterned onto regions of the substrate both with higher and lower doping, while other regions of the substrate were left unpatterned. We will call these four regions higher- and lower-doped substrate and structures, respectively. Native oxide layers (SiO_2_) are also present on the surface. Further details of the fabrication process can be found in Materials and Methods.

As shown in Fig. 1A, in our reflectometer, both the sample and the EUV charge-coupled device (CCD) camera can be rotated about the focused illumination beam to image the sample at incidence angles ranging from ~10° to 60° from grazing. In this experiment, we recorded a series of five high dynamic range (HDR) ptychographic images of the sample, one at each incidence angle from 21° to 25° (measured from the sample surface), in 1° increments. In addition, a later scan was recorded with a slightly different FOV (shifted by ~20 μm) and angle (30°) for improved visualization.

The phase images from variable-angle ptychographic imaging were first coregistered and then segmented to measure phase steps within the FOV as a function of the incidence angle: the step between the higher-doped structures and the lower-doped substrate and the phase step between the higher- and lower-doped substrate. Pixels within each region were averaged to improve the signal-to-noise ratio (SNR) of the calculated phase steps. Then, to solve for the depth-resolved composition reconstruction, we modeled the sample with the parameters of interest varied about their nominal values (e.g., layer thicknesses, composition, etc.). We then used the Parratt formalism (*40*) to calculate the complex reflectance of our candidate stacks and iteratively refined parameter estimates with a genetic algorithm that attempted to match the calculated phase steps from the candidate stacks to the measured phase steps. This allowed us to solve for the depth-resolved chemical composition of the sample and experimental self-calibration parameters. More information about the genetic algorithm implementation can be found in the Supplementary Materials.

## RESULTS

### 3D nanostructure mapping using phase-sensitive imaging reflectometry

The first step in the image processing pipeline was to reconstruct a series of ptychographic images of the sample, one at each incidence angle. It is important for these reconstructions to be accurate, since the composition reconstruction is based on the complex reflectances obtained in this step. Figure 1 (B to D) shows the result after several new data preprocessing and image reconstruction procedures were used to increase the fidelity of the images. First, as our microscope is in reflection mode, the diffraction patterns collected at near-grazing angles were interpolated onto a linear spatial frequency grid through a process we call tilted-plane correction (*41*, *42*). We have found that careful implementation of this process (accommodating all three rotation angles of the sample) with accurate parameters is critical for good image fidelity, especially when imaging at a near-grazing angle. Second, we have incorporated TV regularization (*39*) in the reconstruction algorithm to help remove noise and artifacts by favoring solutions with sparse gradients, which is a good assumption for our sample types. Figure 1 (B and C) shows reconstructions of the same dataset, with and without these improved procedures, respectively. Note that the image after improvements has sharper structure edges and higher fidelity and no longer has the skew seen in Fig. 1B. Figure 1D shows the full reconstruction after the improvements. High-fidelity features are reconstructed far beyond the positions of the center of the scanned EUV beam, which roughly corresponds to the FOV shown in Fig. 1C, and even beyond the region encompassing 99% of the photons accumulated over the full scan.

**Fig. 2:**
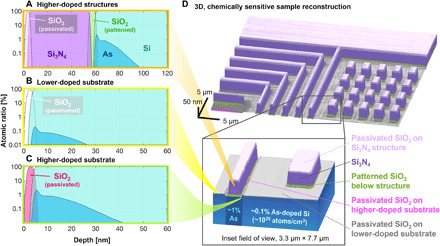
Spatially resolved, composition-sensitive, 3D nanostructure characterization. Composition versus depth reconstruction in the (**A**) higher-doped structures, (**B**) lower-doped substrate, and (**C**) higher-doped substrate. The phase-sensitive imaging reflectometer has sensitivity to most parameters within this model (including layer thicknesses and the dopant concentration). Some parameters were determined by correlative imaging (such as surface roughness and interface diffusion). (**D**) Zoom-out and zoom-in (inset) of fully reconstructed sample. This combines the segmented high-fidelity ptychography reconstruction with the material reconstruction from the genetic algorithm, thus showing spatially and depth-resolved maps of material composition, doping, and topography. Different colors correspond to different materials. Notably, different regions of SiO_2_ are colored uniquely: patterned SiO_2_ under the structures, passivated SiO_2_ on higher- and lower-doped substrate, and passivated SiO_2_ on top of the structures. Also note that we reconstruct the etching adjacent to wide grating lines, shown in magenta in the inset.

Once we had a series of images of the sample’s complex reflectance, we segmented the images to calculate the phase steps between different regions of the sample as a function of incidence angle, and using these, we solved for the depth-dependent material composition of the sample. Figure 2 highlights the capability of the phase-sensitive imaging reflectometer to nondestructively map, in three dimensions, the chemical composition of general samples. Our reconstructions of the composition versus depth of different nanopatterned regions (the higher-doped structure and higher- and lower-doped substrate) are shown in Fig. 2 (A to C). Further parameters that we reconstructed, as well as the calculated sensitivity to those parameters, are listed in Table 1. These can be categorized as parameters related to layer thickness, interface quality, and dopant concentration (as well as experiment self-calibration parameters, shown in the Supplementary Materials).

**Table 1:** Sensitivity of the phase-sensitive imaging reflectometer. This table compares the reconstructed values of different sample parameters by multiple metrology techniques. The “nominal value” column contains the design parameters. For phase-sensitive imaging reflectometry, the “Simultaneous” column shows the values simultaneously solved for using the genetic algorithm with the experimental data; only some of the sample parameters were solved for because of the limited number of data points. Were images at more angles available, we expect that we could simultaneously solve accurately for more of these parameters. The “single-parameter” column shows the sensitivity to these parameters in a single dimension, measured by how much the fit to the data worsens if an individual parameter is varied around the found solution. This column is a rough estimate of how low the confidence intervals could get with this dataset if we were solving for fewer parameters and were able to fix the rest using other metrology techniques. The error bars in the phase-sensitive imaging reflectometry columns are given at 1 SD, while the ranges reported for other techniques, when given, are more loosely defined reasonable ranges given to each measurement. For single-parameter confidence interval calculation, the dopant concentration versus depth was parameterized as the concatenation of an exponential spike at the surface and a Gaussian extending into the bulk (see the Supplementary Materials for complete table).

**Feature**		**Nominal value**	**Phase-sensitive imaging** **reflectometry**		SIMS*	**AFM**	**EDS/HAADF**
			**Simultaneous**	**Single-** **parameter** **confidence** **interval**			
Layer thickness [nm]	SiO_2_ on Si_3_N_4_ structure	0–4	(Set to 3)	± 0.3	–	–	3.0–5.0^†^
	Si_3_N_4_ in structure	50	(Set to 50)	Lower bound: 30	–	–	41–45
	Patterned SiO_2_ under structure	5	(Set to 5)	No sensitivity at 30-nm wavelength	–	–	6.5–7.5
	Structure height	–	48.2 ± 0.2	± 0.02	–	45.0–45.8	48–51
	SiO_2_ on higher- doped substrate	0–4	2.7 ± 0.3	± <0.05	–	–	2.0–4.0^†^
	SiO_2_ on lower-doped substrate	0–4	2.0 ± 0.3	± <0.05	–	–	2.0–4.0^†^
	Dopant-related etch depth	–	6.09 ± 0.07	± 0.02	–	7.8–8.0	5.5–7.5
Interface quality [nm]	Average surface/ interface roughness	–	(Set to 0.5)	Upper bound: 0.8	–	–	0.5–1.0
	Surface roughness on structures	–	(Set to 0.5)	± 0.2	–	0.4–0.5	–^†^
	Surface roughness on lower-doped substrate	–	(Set to 0.5)	± 0.1	–	0.4–0.5	–^†^
	Surface roughness on higher-doped substrate	–	(Set to 0.5)	± 0.3	–	0.4–0.5	–^†^
Dopant	Depth-integrated dose [atoms/cm^2^]	1.10 × 10^15^	0.75 × 10^15^ Upper bound: 5.6 × 10^15^	Upper bound: 2.1 × 10^15^	1.05 × 10^15^	–	1.30 × 10^15^
	Peak concentration [atomic %]	–	(Shape set by SIMS)	Upper bound: 9.3	3.8	–	3.1–4.1
	Gaussian height [atomic %]	–	(Shape set by SIMS)	Upper bound: 3.2	1.1	–	0.8–1.8
Technique summary	Topography		Model-based		–	Direct	Direct
	Composition information		Model-based		Spectroscopic	–	Spectroscopic
	Depth information		Model-based		Direct	–	Direct
	Transverse spatial resolution		Nano-scale (10–100 nm)		TOF/nano-SIMS: ≥ 100 nm	Nano-scale (10–100 nm)	Atomic scale (1–100 Å)
	FOV		Meso-to-micro (10–1000 μm)		–	Meso (10 nm- 100 μm)	Atomic-to-nano (1–1000 nm)
	Sample preparation		Minimal		Minimal	Minimal	Versatile challenging
	Destructive		Non-destructive		Destructive	Contract-based	Destructive

* The dopant measurements by SIMS were taken on an unpatterned sister wafer. The technique could have made similar measurements on layer thicknesses if there were wafers with the same fabrication steps as this sample, but with much bigger feature sizes (size depends on instrument).

† Variation in the SiO_2_ thicknesses between phase-sensitive imaging reflectometry (i.e., with phase and amplitude sensitivity) and EDS/HAADF is expected, because the sample had sufficient time to oxidize further between the two measurements. The sample was not prepared to perform surface roughness measurements.

**Fig. 3:**
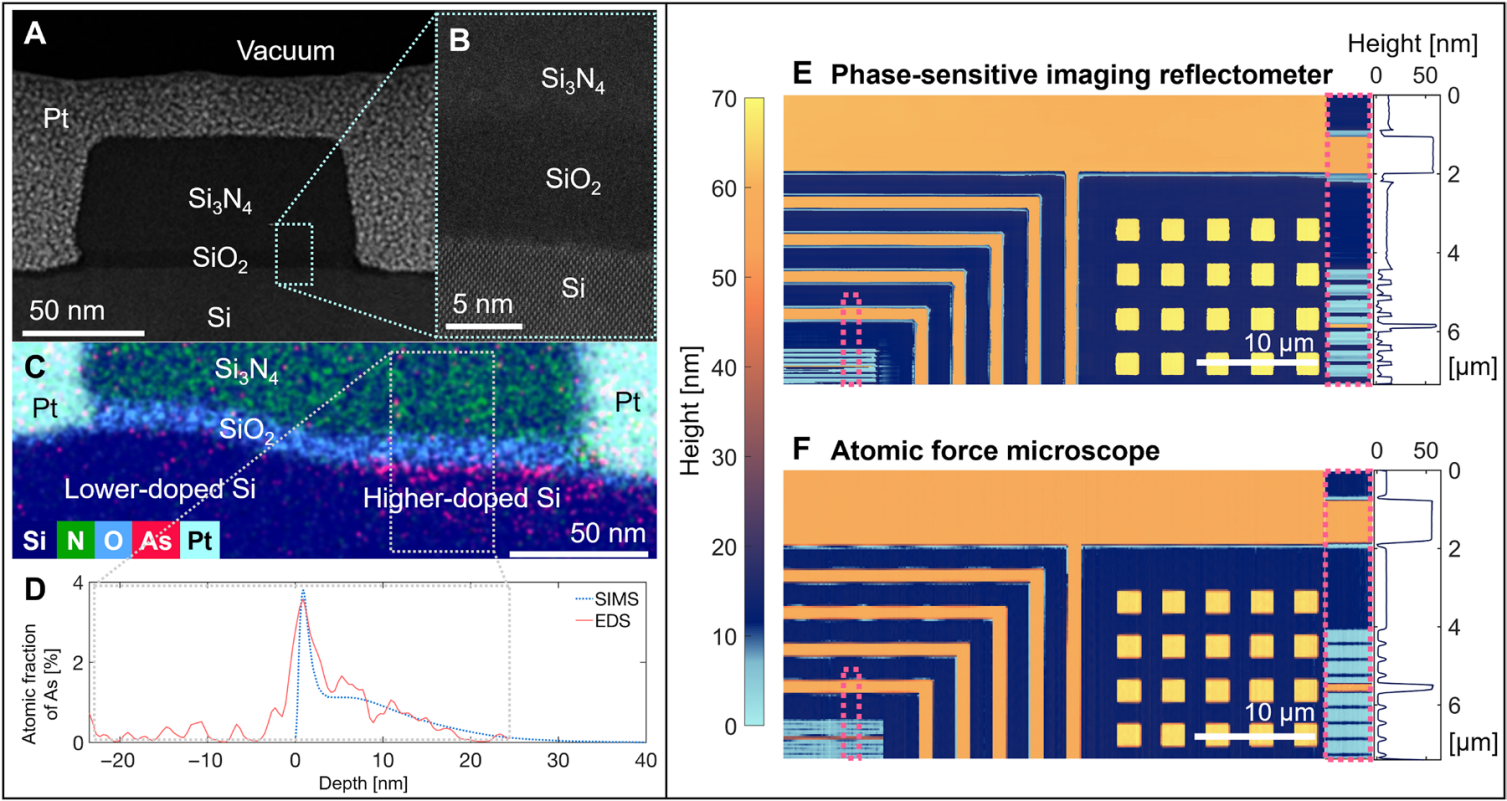
Correlative imaging with TEM and AFM. (**A**) High-angle annular dark-field (HAADF)–STEM image of one of the Si_3_N_4_ structures prepared by focused ion beam (FIB), with (**B**) a zoom-in showing the interfaces between Si, SiO_2_, and Si_3_N_4_. (**C**) An EDS image showing a different Si_3_N_4_ structure that is doped to ~1% on the right half and to ~0.1% on the left half. (**D**) EDS dopant-versus-depth profile that compares well to the curve obtained using SIMS measured on an unpatterned wafer. To increase the SNR, the energy-dispersive x-ray spectroscopy (EDS) profile was integrated over the area marked by the gray dotted box in (C). (**E**) Topography map obtained by combining the ptychographic phase image with the results of the genetic algorithm. The pixel size is 64 nm × 172 nm (vertical × horizontal), and the axial precision is 2 Å. (**F**) AFM image of the same region. Zoom-in on a region and averaged lineouts of that region are shown on the right.

Figure 2D shows the topographic and material map produced by the reflectometer. The different colors in Fig. 2 correspond to materials treated differently in the genetic algorithm. For example, there are four colors that each represent a different region of SiO_2_ (patterned SiO_2_ under the Si_3_N_4_, passivated SiO_2_ on top of the Si_3_N_4_, and passivated SiO_2_ on the higher- and lower-doped substrate). To produce this figure, the complex reconstruction whose phase is shown in Fig. 1C was segmented into the four regions of different compositions using *k*-means clustering, and each segmented region was rendered with the topography and composition solved for by the genetic algorithm (Figs. 3E and 2, A to C, respectively). Slight, per-pixel deviations from the resulting height of each region were calculated from the small deviations in the phase from the average phase within the corresponding region.

### Correlative imaging of the nanostructure

We generated a height map (Fig. 3E) in the same way as the material map (Fig. 2D) to compare with the topography from the AFM measurement. We estimate that the resolution in this image is comparable to the pixel size defined by the detector NA, which is 64 × 172 nm (vertical × horizontal). We have two independent observations supporting this resolution. First, the presence of substantial diffraction intensity extending to the vertical and horizontal edges of the detector suggests spatial frequency content that fills that bandwidth. Second, evaluating this resolution from the reconstruction itself, we note that the dopant-related etching seen immediately around the structures in Fig. 3 (E and F) is ~120 nm wide (as measured by AFM). Since this etching has a complex-valued reflectance that is not between the reflectance values of the neighboring regions on either side, this reconstructed feature is not a blurring artifact and serves the purpose of acting as a resolution-test target. The fact that we see the etching in both directions with enough visibility for the generic *k*-means clustering algorithm to properly segment the image indicates that we have a resolution approaching or surpassing 120 nm in each direction. However, while this is supported by the detector NA in the vertical direction, it is unexpected that we see these features even in the horizontal direction, where the etch width is slightly smaller than a single pixel; we expect that this is accompanied by a reduction in visibility of the etching gap. Therefore, from the above two considerations, we conclude that the resolution is comparable to the pixel size defined by the detector NA. The anisotropy in resolution comes from conical diffraction, which stretches the diffraction pattern in the direction of incidence, improving sampling and FOV but reducing the resolution (*43*).

Because no other single technique could verify all the parameters that we solved for with phase-sensitive imaging reflectometry, we used several correlative imaging techniques on identical copies of the sample to validate our extracted sample parameters. These techniques included SIMS on an unpatterned sister wafer As-doped to ~1% (Fig. 3D), both high-angle annular dark-field STEM (HAADF-STEM) and energy-dispersive x-ray spectroscopy (EDS) (Fig. 3, A to C) on a small cross section of the sample and AFM (Fig. 3F). Note that the first three techniques are destructive, requiring focused ion beam (FIB) milling of the sample, and HAADF-STEM and EDS require special sample preparation. The results of these techniques and phase-sensitive imaging reflectometry are compared in Table 1. The two columns related to phase-sensitive imaging reflectometry outline the promising results of this technique, both when solving for many parameters simultaneously and when solving only for a single parameter (i.e., if the other parameters are known a priori or measured by other techniques). To avoid overfitting, we restricted the composition reconstruction to solve only for as many unknowns as we had measured phase steps, so the fixed values in the “simultaneous” column result from the limited number of angles in this initial series of measurements. The values shown in the single-parameter column are intended to give an idea of the order-of-magnitude sensitivity of 30-nm light to each sample parameter. These numbers should not be taken as estimates of the best error bars achievable when solving for a certain parameter: For datasets with more measurements or better-selected wavelength, the error bars could be much better than those shown, and averaging a greater number of pixels in the segmentation step can also improve these values. In general, we find that phase-sensitive imaging reflectometry has good agreement with other techniques and is sensitive to many sample parameters that are crucial for nanomaterials.

## DISCUSSION

The phase-sensitive imaging reflectometry approach introduced and demonstrated here is extremely versatile and general. It is sensitive to almost all the sample parameters shown in Table 1 and has the capability to reconstruct all these in a highly spatially and depth-resolved manner, nondestructively, without contact, and without special sample preparation.

In general, the sensitivity of this technique primarily depends on how many photons can reach and scatter off of the feature of interest. For instance, 29.3-nm light has a penetration depth of approximately 30 nm in Si_3_N_4_ (*14*), so solving for layer thicknesses of 1 to 10 nm works extremely well. However, features that lie beneath the 50-nm structures in this sample were more difficult to extract, since at 25° and in reflection, the EUV photons need to pass through 2 × 50/sin(25°) ≈ 250 nm of material. Using either shorter wavelength ~13-nm light that is more penetrating or incidence angles farther from grazing would substantially enhance the ability to detect deeper buried features. Moreover, the sensitivity to interface roughness is proportional to (σ/λ)^2^Δ*_n_*, where σ is the surface roughness, λ is the illumination wavelength, and Δ*_n_* is the difference in the index of refraction of the two layers (see the theory section in the Supplementary Materials). Since the surface roughness in this sample is much smaller than 30 nm, the interfaces in this sample represent a challenge to probe using 30-nm light. In the future, by using 13-nm illumination, the sensitivity to interfaces will improve by making (σ/λ)^2^ more favorable, while Si_3_N_4_ is ~4× more transparent to 13-nm light (penetration depth of 125 nm) (*14*), so that more photons can reach buried interfaces.

In this demonstration, it was shown that this technique can solve for layer thicknesses, dopant levels, and experiment self-calibration (see the Supplementary Materials). Layer thicknesses, dopant level, and interface quality (to which the single-parameter column in Table 1 shows promising sensitivity) are crucial for proper function of many modern semiconductor, quantum, and magnetic devices. With our technique, we were able to set an upper bound on the dopant concentration. We used SIMS to set the shape of the dopant profile and used our data to solve for the dopant concentration. Promisingly, the single-parameter sweep column of Table 1 shows that, even with this preliminary dataset, we have sensitivity to the shape of this curve. Thus, we expect that either with measurements at more incidence angles or by using 13-nm light, this technique should have sufficient element sensitivity to resolve this curve’s shape natively.

The self-calibration capability allows for more accurate measurement of quantities that are difficult to precisely determine (such as the absolute incidence angle of the illumination on the sample, arising from imperfect sample mounting). By solving for these parameters in the same step that performs the composition reconstruction, we jointly optimize the sample parameters and microscope calibration and become robust to errors in the system alignment. Furthermore, this system does not suffer as much as other 3D techniques (e.g., tomography) from stringent requirements on physical system alignment that are required to have exactly registered FOVs, since the images are reconstructed independently and can be registered (manually or automatically) after the fact.

We note that the ability to measure dopants in nanodevices is mostly limited to destructive techniques. Auger electron spectroscopy and SIMS are able to measure dopant levels but are destructive and typically restricted to unpatterned samples [with a few notable exceptions (*44*, *45*)]. On the other hand, TEM-based techniques such as off-axis electron holography, EDS, and electron energy-loss spectroscopy can measure dopant concentrations in nanostructures with high spatial resolution, but again are destructive, have challenging sample preparation, and are inherently localized techniques with limited FOV (*46*–*48*).

Model-based scatterometry techniques share some similarities with phase-sensitive imaging reflectometry (*49*), but our technique has unique advantages. Scatterometry-based techniques start from an informed, 3D model of the sample and solve for sample parameters. While the last step is similar, we can reconstruct the sample without a detailed knowledge of its transverse structure and require only a 1D model in depth, which requires minimal knowledge of the fabrication steps. In the case that there are uninteresting regions of the sample that are unknown or difficult to model (specs of dust, etc.), our model-free 2D reconstruction can reveal them, and we can avoid them easily by simply omitting those pixels from the depth reconstruction. In contrast, such a feature would often negatively influence the data and the outcome of most scatterometry-based techniques without the user knowing, and even if the user notices the presence of the unexpected feature, then they would need to turn to an imaging technique for further information.

Last, we note that a unique advantage of this new technique is its ability to tune the error bars, after the data are already taken, to the sensitivity required to detect a given feature. By selecting larger regions of interest in the image segmentation, the reconstructed composition versus depth can be improved at the cost of transverse spatial resolution. Of course, there is a limit to the SNR achievable once a dataset is taken, but this ability to tune, almost continuously, the balance of SNR and spatial information to detect a desired feature of interest is remarkable. In high-quality datasets, this could allow one to reconstruct a depth profile for every pixel or structure in the FOV, forming a rich 3D map of the sample’s composition, topography, and interfaces.

We have developed a unique and versatile phase-sensitive imaging reflectometry technique that can nondestructively map the depth-dependent composition of materials, as well as nanostructure layer thicknesses and interface quality, all in a highly spatially resolved manner. Our results demonstrate that EUV phase-sensitive imaging has exquisite profile sensitivity. By combining the unique strengths of tabletop, coherent, EUV high-harmonic sources with excellent phase stability and CDI, we can address imaging science challenges associated with the synthesis and integration of next-generation quantum, semiconductor, and spintronic devices and heterostructures, independent of architecture. In the future, it will be possible to enhance the chemical/topographic contrast and the spatial resolution (to <10-nm transverse resolution and <1-Å axial precision) by using shorter- or multiwavelength illumination and by imaging the sample at multiple in-plane rotational orientations and/or higher NA. Moreover, by harnessing the femtosecond time resolution of EUV HHG beams, the imaging reflectometer can be enhanced further to capture charge and spin and heat transport in the next-generation devices and link structure to function. Thus, this work represents a fundamentally new and useful approach for imaging nanostructures and nanomaterials that has unique advantages compared to complementary techniques such as electron, atomic force, and other scanning probe microscopies.

## MATERIALS AND METHODS

### Experimental design

To generate the 29.3-nm HHG beam used to illuminate the reflectometer, we focused a femtosecond Ti:sapphire laser (2.1 W, 0.7 mJ, 35 fs, 3 kHz, 0.79 μm) into an argon-filled hollow-core waveguide (150-μm inner diameter, 30-torr Ar). The resulting flux in the 29.3-nm harmonic was ~10^12^ photons/s at the source, with <1% root mean square (RMS) power stability. This corresponds to 3 × 10^9^ photons/s at the sample, for the nonoptimized beamline used for this initial demonstration experiment. Although ptychographic imaging is robust to noise in the recorded data, it does require that the illumination beam be very stable in intensity, wavelength, and wavefront over the course of the scan (*50*, *51*). Thus, in our HHG setup, we ensure that we have a very good driving laser stability in both pointing and intensity (0.85% RMS), as well as optimal phase matching conditions.

The residual driving laser light was filtered out using two Si rejector mirrors oriented near Brewster’s angle for infrared, followed by two 200-nm-thick Al filters. A single HHG order was then selected using a pair of SiC/Mg multilayer mirrors. This beam was focused onto the sample using a grazing-incidence ellipsoid, to a spot size of 10 μm × 10 μm at normal incidence for the high-fidelity image and 21 μm × 21 μm for the imaging reflectometry datasets used for composition reconstruction. Note that a 10-μm-diameter focus elongates to as wide as 28 μm at the grazing angles of incidence used in this work. A wavelength of 29.3 nm was used in this initial demonstration to take advantage of the relatively higher reflectivity of the sample at longer wavelengths: The S-polarized reflectivity of passivated Si at angles between 21° and 25° from grazing is 3 to 15%.

We recorded a series of ptychographic datasets using a 29.3-nm HHG beam as the illumination, with 4 × 10^7^ photons/s incident on the sample. We collected five ptychographic scans on the sample at incidence angles between 21° and 25° (measured from the sample surface), in 1° increments. Each ptychographic scan in the imaging reflectometry dataset contained 301 positions in a Fermat spiral configuration (*52*). Two exposures were collected at each beam position on the sample for HDR. The lower exposure time at each angle was set 10% shorter than that required to saturate the brightest pixels at that angle, and the longer time was twice of that. Before and after data collection at each angle, 150 frames were recorded with the HHG beam pointing directly on the camera (by moving the sample out from the beam and rotating the camera such that the beam is normally incident on the sensor), and background images (with the beam blocked from the system) were recorded for scan and beam data with 0.75-s exposure times. We used the resulting ptychography reconstructions to perform the material reconstruction. These images are shown in the Supplementary Materials, as is information about additional scans not discussed here.

In addition, a later ptychographic dataset was recorded with a slightly different FOV (shifted by ~20 μm) and angle (30°) for improved visualization. This dataset had slightly different parameters. An iris was introduced before the harmonic selecting multilayer mirrors to add structure to the out-of-focus EUV beam. The scan pattern was a rectangular grid instead of a Fermat spiral and consisted of 424 scan positions (non-HDR), each with an exposure time of 0.1 s. Background data were also recorded with 0.1-s exposure times. We also took beam data before and after the main ptychography scan. These frames were used for the implementation of the modulus enforced probe constraint (*4*) on the ptychographic reconstruction of the beam. The resulting image of the sample was not incorporated into the material reconstruction because it was taken with a different system alignment than the imaging reflectometry scan, and would introduce almost as many uncertainties (exact incidence angle and wavelength) as it helped solve for. All the data were collected with 1 × 1 binning and a 1-MHz readout rate on a Princeton Instruments MTE2 CCD (2048 × 2048, 13.5-μm pixels).

### Sample fabrication

The fabrication process of the sample studied in this work is illustrated in fig. S11. First, select regions of the Si wafer were doped with As (5-keV ion implantation) with dose of 10^15^ atoms/cm^2^. Then, the photoresist masking the other regions was stripped off, and the entire sample was doped with As with dose of 10^14^ atoms/cm^2^. This created patterns of higher- versus lower-doped regions on the Si substrate. When the photoresist was stripped, however, it also caused the surface of the unprotected higher-doped regions to be partially removed, creating an unintended dishing of the higher-doped substrate with respect to the lower-doped substrate. The substrate was spike annealed at 1035°C. Then, nominal 5 nm of SiO_2_ and then 50 nm of Si_3_N_4_ were deposited everywhere on the surface of the sample. Photoresist was then patterned on top of these layers, and the unprotected regions were etched away, creating protruding surface structures. This process resulted in thicker deposited SiO_2_ underneath the structures, compared to the passivated SiO_2_ on top of the substrate. There is also passivated SiO_2_ on top of the Si_3_N_4_ structures.

### Topography and composition rendering

The process of creating the composition-sensitive 3D rendering shown in Fig. 2D involved several processes that ultimately incorporate both the ptychographic reconstruction of the sample and the composition and topography parameters that were solved for in the composition reconstruction.

In general, the phase in ptychographic reconstructions is influenced by both the chemical composition and the surface topography. Within each of the four different regions on our sample (higher- and lower-doped substrate and structures), the mean phase was assumed to come from the average height and the chemical composition, while the variation from the mean was assumed to come from small local topographic variations. Therefore, the ptychographic reconstruction had to be first segmented into the four regions. To do so, first, the phase reconstruction was unwrapped to remove any remaining linear phase ramps in the image so that the phases may be physically interpreted. Then, to accurately render the composition-coded color map for all surfaces (including sidewalls of structures), both the phase and amplitude reconstructions were upsampled by a factor of two using modified Akima interpolation (*53*). To produce a map identifying and labeling the four distinct regions of the reconstruction, this complex image was segmented with a *k*-means algorithm using the amplitude and phase images as the only two channels. To improve robustness of the segmentation, the region containing the fine grating was segmented independently of the remaining part of the sample.

The map of labels produced by the segmentation was then refined through two filtering steps, each performed on separate sections of the reconstruction. The square structures located in the lower right of the image were processed with a binary filter, and the narrow grating located in the lower left was processed with a median filter. In addition, a very small cluster of clearly mislabeled pixels (likely due to ringing artifact) was manually relabeled. In all, these filtering steps only affected less than 0.7% of the pixels in the image and resulted in label maps that more closely resembled the regions one would identify by eye in the phase and amplitude maps. The remainder of the image did not require any filtering or additional processing.

After the ptychographic reconstruction was segmented into four regions, the average phase of each region was subtracted from the phase map to find the variation from average for every point. This relative phase map was converted to a relative height map by an incidence angle–dependent factor [*h =* Φ/2 *k* sin(θ), where *h* is the height, Φ is the phase in radians, *k* is the wave vector, and θ is the angles from grazing]. The relative height map was then combined with the step heights solved in the composition reconstruction to determine the absolute height of each point in the rendering. After the surface topography was fully determined, independently for each region, composition color coding was performed using the layer thicknesses solved in the composition reconstruction.

### Sensitivity analysis

To calculate the confidence interval on each of the fitted parameters, we follow a method that uses the covariance matrix, as described by Press *et al.* (*54*). To characterize the curvature of the error metric landscape around the found solution, a matrix of double derivatives at that point can be calculated. To an approximation, the elements of this matrix **α** (referred to as the curvature matrix, or one-half of the Hessian matrix) can be expressed using single derivatives

$$\alpha_{\mathrm{kl}}=\overset{N}{\sum_{i=1}}\frac{1}{\sigma_{i}^{2}}[\frac{d\varphi (\theta_{i}|a)}{{\mathrm{da}}_{k}}\frac{d\varphi (\theta_{i}|a)}{{\mathrm{da}}_{l}}]$$ (1)

where φ are the measured phase steps, φ*(*θ*_i_*|***a***) are the calculated phase steps for the corresponding data point with incidence angle θ*_i_* and vector of solved-for parameters ***a***, and σ*_i_* is the standard error of the mean (SEM) for that data point.

The single derivatives can be approximated numerically using the equation below (higher-order methods can be used, but it was found that the discrepancy is negligible)

$$\frac{d\varphi (\theta_{i}|a)}{{\mathrm{da}}_{k}}=\frac{\varphi (\theta_{i}|+\Delta a_{k})-\varphi (\theta_{i}|-\Delta a_{k})}{2\Delta a_{k}}$$ (2)

where φ(θ*_i_*| + Δ*a_k_*) is the phase step calculated at the found solution but with the *k*th parameter displaced by step +Δ*a_k_*.

Once the curvature matrix has been numerically calculated, its inverse known as the covariance matrix ***C*** is obtained, and the confidence interval δ*a_k_* on the *k*th solved parameter is found using its diagonal elements

$$C=\alpha^{-1}$$ (3)

$$\delta a_{k}=\pm \sqrt{\Delta \chi^{2}}\sqrt{C_{\mathrm{kk}}}$$

where Δχ^2^ for the desired confidence level can be looked up from a reference table (*54*). The confidence intervals reported in the “Phase-sensitive imaging reflectometry, simultaneous” columns in Table 1 and table S1 were calculated using this method, for 1-SD confidence interval.

In addition to the confidence intervals calculated for when parameters were solved simultaneously using a genetic algorithm, we also report the “single-parameter” confidence intervals in a single dimension, measured by how much a parameter can be varied, while the other parameters are fixed, before the error increases by Δχ^2^ = 1 (*54*). This column is a rough estimate of how low the confidence intervals could get with this dataset if we were solving for fewer parameters and were able to fix the rest using other metrology techniques.

Using the off-diagonal elements of the covariance matrix and the confidence intervals on the parameters, the correlation coefficient *r* between the *k*th and the *l*th solved-for parameters can also be calculated, using the equation below (*54*)

$$r_{\mathrm{kl}}=\frac{C_{\mathrm{kl}}}{\delta a_{k}\delta a_{l}}$$ (4)

**Fig. 4:**
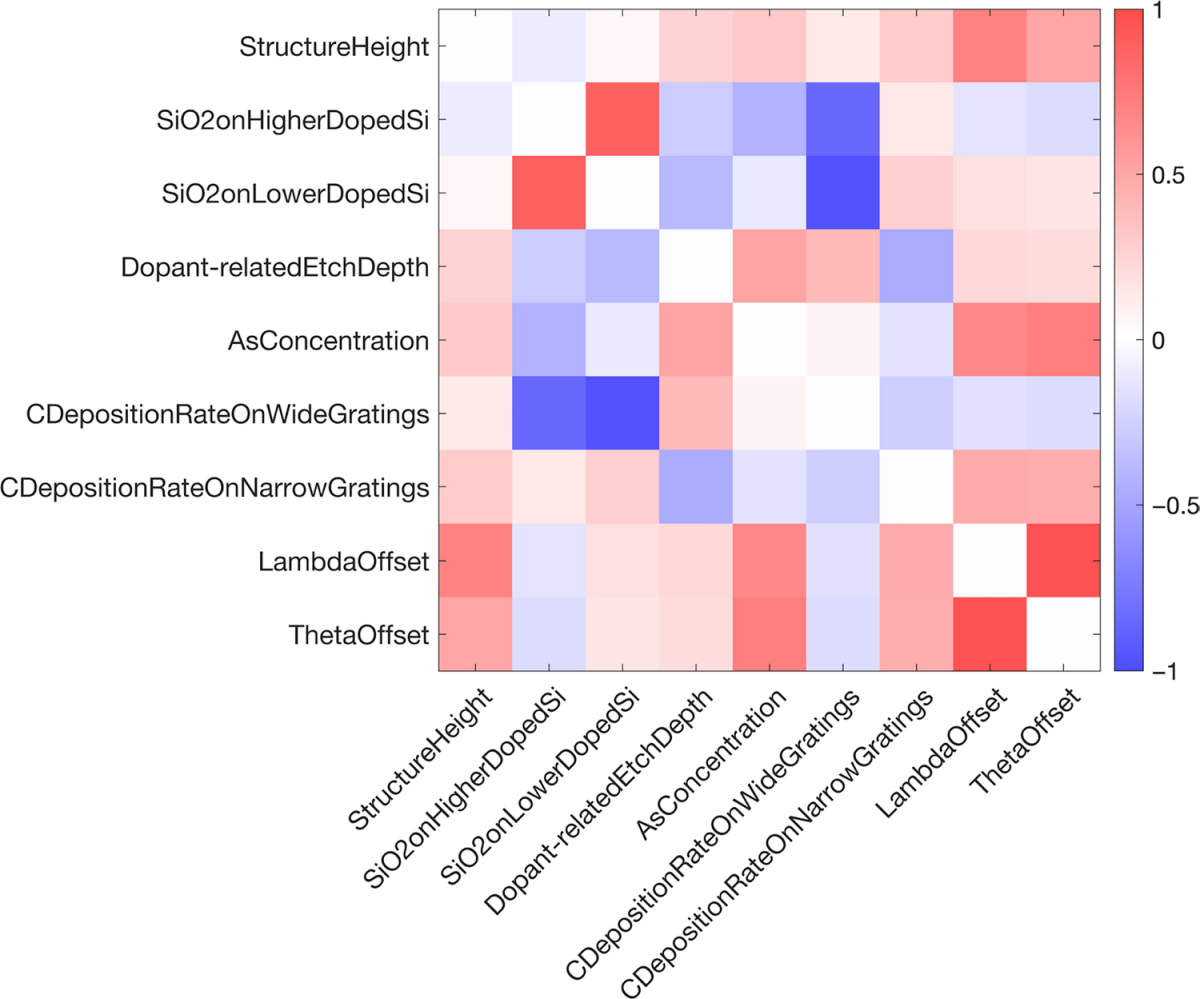
Correlation coefficients between the nine sample parameters in the composition-versus-depth reconstruction.

Figure 4 shows the correlation coefficient between all the nine parameters solved for using the genetic algorithm. High magnitude of correlation coefficient means that increase in error due to change in one parameter can be well compensated by change in the other parameter, so it is favorable for correlation coefficients to have low magnitude. In the plot, there are only three parameter pairs that have correlation coefficient >0.85. Given the strong correlation between the wavelength and the incidence angle offsets, it may have been preferable to fix one of them to the nominal value of zero and only solve for the other, or solve for some factor that combines the two. It is unclear why there is high correlation between SiO_2_ thickness on higher- and lower-doped substrate and the carbon deposition rate on wide gratings FOV and the SiO_2_ thickness on lower-doped substrate; it is possible that this experiment was more sensitive to the difference, or the ratio, of the thicknesses of the layers concerned.

## SUPPLEMENTARY MATERIALS

Supplementary material for this article is available at http://advances.sciencemag.org/cgi/content/full/7/5/eabd9667/DC1
